# Role of Active Contraction and Tropomodulins in Regulating Actin Filament Length and Sarcomere Structure in Developing Zebrafish Skeletal Muscle

**DOI:** 10.3389/fphys.2016.00091

**Published:** 2016-03-31

**Authors:** Lise Mazelet, Matthew O. Parker, Mei Li, Anders Arner, Rachel Ashworth

**Affiliations:** ^1^School of Biological and Chemical Sciences, Queen Mary, University of LondonLondon, UK; ^2^School of Health Sciences and Social Work, University of PortsmouthPortsmouth, UK; ^3^Department of Physiology and Pharmacology, Karolinska InstitutetStockholm, Sweden; ^4^The Blizard Institute/Institute of Health Sciences Education, Barts and The London School of Medicine and DentistryLondon, UK

**Keywords:** skeletal muscle, zebrafish, tropomodulin, myofibrils, contraction, active and passive tension

## Abstract

Whilst it is recognized that contraction plays an important part in maintaining the structure and function of mature skeletal muscle, its role during development remains undefined. In this study the role of movement in skeletal muscle maturation was investigated in intact zebrafish embryos using a combination of genetic and pharmacological approaches. An immotile mutant line (*cacnb1*^*ts25*^) which lacks functional voltage-gated calcium channels (dihydropyridine receptors) in the muscle and pharmacological immobilization of embryos with a reversible anesthetic (Tricaine), allowed the study of paralysis (in mutants and anesthetized fish) and recovery of movement (reversal of anesthetic treatment). The effect of paralysis in early embryos (aged between 17 and 24 hours post-fertilization, hpf) on skeletal muscle structure at both myofibrillar and myofilament level was determined using both immunostaining with confocal microscopy and small angle X-ray diffraction. The consequences of paralysis and subsequent recovery on the localization of the actin capping proteins Tropomodulin 1 & 4 (Tmod) in fish aged from 17 hpf until 42 hpf was also assessed. The functional consequences of early paralysis were investigated by examining the mechanical properties of the larval muscle. The length-force relationship, active and passive tension, was measured in immotile, recovered and control skeletal muscle at 5 and 7 day post-fertilization (dpf). Recovery of muscle function was also assessed by examining swimming patterns in recovered and control fish. Inhibition of the initial embryonic movements (up to 24 hpf) resulted in an increase in myofibril length and a decrease in width followed by almost complete recovery in both moving and paralyzed fish by 42 hpf. In conclusion, myofibril organization is regulated by a dual mechanism involving movement-dependent and movement-independent processes. The initial contractile event itself drives the localization of Tmod1 to its sarcomeric position, capping the actin pointed ends and ultimately regulating actin length. This study demonstrates that both contraction and contractile-independent mechanisms are important for the regulation of myofibril organization, which in turn is necessary for establishing proper skeletal muscle structure and function during development *in vivo* in zebrafish.

## Introduction

The myofibrils of muscle contain myofilament proteins (e.g., actin and myosin) which constitute the building blocks of the sarcomere, the contractile unit of striated muscle. Myofibrils which run in parallel contain repeating sarcomeres organized in series. The mechanical function of skeletal muscle is in part determined by the spatial arrangement of muscle fibers relative to the axis of force generation (Bian et al., 2012), and the changes to muscle fiber architecture, such as disorganization of myofibrils or fibrosis which can affect force generation. Furthermore, muscle structure adapts to functional constraints, so that alterations in stretch or force production influence both muscle mass and phenotype (Goldspink, 1998; Saladin, 2010). Muscle fiber organization at the myofibril level is an important component of co-ordinated contraction; however, the mechanisms through which this complex subcellular architecture is established and maintained, especially during development, is only partially understood. Knowledge regarding this process would have an impact on our understanding of muscle dysfunction in humans and potentially for developing novel therapeutic strategies.

Mechanotransduction plays a key role in maintaining the structure of mature striated muscle; however, its function during development is much less defined (Boonyarom and Inui, 2006). Evidence from cultured muscle fibers suggests that contraction has a role in the later stages of myofibril assembly, alignment and sarcomere spacing (De Deyne, 2000; Sanger et al., 2005, 2010; Fujita et al., 2007; Sparrow and Schöck, 2009). These *in vitro* studies are supported by *in vivo* work which has revealed that contraction appears important for myofibril development in skeletal muscle developing within intact organisms. In *C. elegans* the final stage of muscle development, which includes sarcomeric organization and the spatial arrangement of cell attachments, coincides with the onset of muscle twitching (Hresko et al., 1994). A stretch-activation pathway that controls several aspects of myofibril assembly has also been identified in *Drosophila* (Reedy and Beall, 1993). In addition, paralysis has been shown to delay Z-disc formation and disrupt thick filament organization in *Xenopus tropicalis* (Geach et al., 2015). A lack of E-C coupling in the developing skeletal muscle of immotile zebrafish mutant lines disrupts myofibril alignment, sarcomere width and filament length (Behra et al., 2002; Brennan et al., 2005; van der Meulen et al., 2005). Thus, in summary, evidence supports the presence of a movement-driven pathway controlling myofibril assembly and maintenance during skeletal muscle development. This appears to be a fundamental mechanism relevant to skeletal muscle development *in vivo* and it is conserved across a variety of species within the animal kingdom (as reviewed in Sanger et al., 2002, 2005).

Studies demonstrating that paralysis disrupts myofibril structure provide indirect support to the idea that there is a movement-driven pathway controlling myofibril assembly and maintenance during skeletal muscle development; however, studies have yet to determine whether contraction can drive myofibril organization directly. Therefore, the main aim of this study was to determine the contribution of contraction to the maintenance of myofibril organization during vertebrate skeletal muscle development *in vivo*. Zebrafish provide several benefits as a model for the study of vertebrate skeletal muscle development, of particular advantage was the availability of mutant lines, their amenability for tissue staining studies and the techniques for assaying behavior and muscle function (Stickney et al., 2000). The role of contraction in the maintenance of myofibril organization was tested by determining whether the restoration of movement after paralysis was sufficient to drive myofibril realignment and recovery of function. The developmental consequences of restoring contraction after immobilization was addressed by examining myofibril structure (at both the myofilament and myofibril level), function (passive and active properties) and swimming behavior. One of the ultimate goals will be to identify key signaling components of the mechano-sensitive pathway that regulates myofibril assembly and maintenance during development *in vivo* as there is very little information in this area. Therefore, this study also examined the effect of paralysis on the localization of the actin capping protein Tropomodulin with the aim of determining which sarcomeric proteins may be involved in movement-driven myofilament disruption.

## Materials and methods

### Zebrafish maintenance

Zebrafish early larvae (*Danio rerio*, Tübingen strain) and the mutant zebrafish line, *cacnb1*^*ts25*^ (*relaxed*), up to 7 dpf, were obtained from either the Karolinska Institutet Zebrafish Facility or Queen Mary University of London Zebrafish Facility and maintained as described previously (Ashworth et al., 2001). Heterozygote adults were crossed to obtain homozygous (rr) embryos, identified by lack of movement after 17 hpf. Embryos from the same batch that displayed a normal phenotype (heterozygote and wild type), were used as controls (RR/Rr, denoted control siblings) (Schredelseker et al., 2005). Stages are given in the text as standard developmental time (hpf) (Kimmel et al., 1995). All animal work was carried out following approval from the Queen Mary Research Ethics Committee, and under license from the Animals (Scientific Procedures) Act 1986 or conformed with the Guide for the Care and Use of Laboratory Animals (1996 National Academy of Sciences, Washington D.C.). Care was taken to minimize the numbers of animals used in this experiment in accordance with the ARRIVE guidelines (http://www.nc3rs.org.uk/page.asp?id=1357). Fish bred and reared in the Zebrafish Facility at Queen Mary University of London were licensed by the UK Home Office.

### Pharmacological immobilization *in vivo*

Dechorionated wild-type embryos were transferred into Embryo Medium (5 mM NaCl, 0.17 mM KCl, 0.33 mM CaCl_2_, and 0.33 mM MgSO_4_, pH 6.8–6.9) supplemented with 0.03% Tricaine (Tricaine methanesulfonate, TMS, MS-222) at 17 hpf, incubated for 7 h at 28.5°C. After 1 h of treatment embryos were examined for locomotor activity, i.e., occurrence of spontaneous movements. Control embryos, treated in exactly the same way as experimental group in Embryo Medium but without Tricaine, were raised in parallel. At 24 hpf, control and treated embryos were fixed and stained immediately or put into recovery (Embryo Medium without Tricaine). The control and recovered embryos were either fixed and stained at 42 hpf or mounted for X-ray diffraction at 5 dpf, or mechanically assessed at 7 dpf or used to assess their swimming behavior (at 3, 5, 7, and 30 dpf). Experiments to measure sarcomere length at different degrees of relative stretch included a group of larvae that were immobilized from 17 hpf to 5 dpf. Embryos were incubated initially at 28.5°C in 0.03% Tricaine from 17 hpf up to 29 hpf, and subsequently the larvae were transferred into 50 μM BTS (N-Benzyl-P-Tuloenesulfonamide, Sigma) and further incubated at 28.5°C up to 5 dpf.

### Mounting for mechanical experiments and X-ray diffraction analysis

The larvae were held in Embryo Medium. Prior to experiment a 4–7 dpf larva was transferred to Embryo Medium containing 0.017% Tricaine, euthanized and the preparation mounted for analysis (Dou et al., 2008; Li et al., 2013) using aluminum clips. These were folded around the head and tail portions of the larva, providing a defined and stable attachment. Small holes were made in the aluminum foil for attaching the preparation between the extended arm of an AE801 force transducer (SensoNor, a.s., Horten Norway) and a fixed pin for length adjustment using a micrometer screw. For mechanical experiments the preparation was held horizontally in a 0.5-ml Perspex organ bath at room temperature (22°C). For X-ray diffraction experiments the preparation was mounted between a fixed hook and a hook on a micrometer screw, for length adjustment. The experiments were performed at 22°C in MOPS buffered physiological solution (in mM: 118 NaCl, 24 MOPS, 5 KCl, 1.2 MgCl_2_, 1.2 Na_2_HPO_4_, 1.6 CaCl_2_, and 10 glucose, pH 7.4).

### Mechanical experiments

The larval preparations were mounted as described above at slack length and then stimulated (single twitches) with 0.5 ms electrical pulses (supramaximal voltage) at 2 min intervals via two platinum electrodes placed on both sides of the preparation. Length (L) was increased in steps between the contractions, from the slack length (Ls) to a length above the maximal (Lopt) for active force. At each length, active and passive force was recorded. Using this procedure the Lopt, as well as the shape of the passive and active length-force relationships, were determined. In one series of experiments we mounted muscle preparations in a cuvette with a glass window enabling analysis of sarcomere length using a 40x objective on an inverted microscope. Muscles were stretched in a similar manner as in the force and X-ray diffraction experiments to different lengths relative to slack. These data enabled us to relate the extent of stretch to sarcomere length.

### Small angle X-ray diffraction

Small angle X-ray diffraction was used to determine the interfilament distances in the zebrafish larval muscle, as described previously (Dou et al., 2008), at beamline I911-SAXS at the MAX II ring of the MAX IV Laboratory in Lund, Sweden (Labrador et al., 2013; Li et al., 2013). The larvae were anesthetized, euthanized and mounted horizontally as described above between two hooks in a Kapton cuvette in the MOPS buffered physiological solution and stretched to different lengths (L) relative to slack (Ls). In a few experiments the MOPS buffered solution was supplemented with 5 mM NaCN to induce metabolic inhibition and a rigor state. The camera length was 230 cm and calibrations of the X-ray patterns were made using diffraction from rat tail collagen. The diffraction patterns were recorded using a PILATUS 1M detector (DECTRIS Ltd.) with exposure times between 5 and 10 s. For analysis of diffraction patterns in living and non-stretched preparations, the larvae were anesthetized with Tricaine and kept in Embryo Medium in a cuvette. Equatorial patterns were recorded and the spacing and intensities of the 1.0 and 1.1 reflections were evaluated using background subtraction by fitting a polynominal function, determining the center position (lattice spacing) and the integrated area (intensity) under the peaks.

### Immunohistochemistry

Embryos were fixed over night at 4°C in BT fix (4% para-formaldehyde, 0.15 mM CaCl_2_, 4% sucrose in 0.1 M PO_4_ buffer), as described previously (Ashworth et al., 2001). The following day, three 10 min washes in phosphate buffer (PB) were performed to remove any excess PFA, followed by a 5 min acetone permeabilisation at −20°C and a further two 5 min washes in PB. A block was then performed using 10% goat serum (GS) in 500 μL phosphate buffer with 0.8% triton (PBT), samples were incubated for 1 h. Where stated primary antibodies were made up in phosphate buffered saline with 0.8% triton (PBST) and 1% GS saline solution and incubated overnight. F59 (DSHB Hybridoma Product F59, raised in mouse) was made up at a concentration of 1:100, Tropomodulin 1 and Tropomodulin 4 (kind gift from Professor Fowler, both raised in rabbit) were made up at a concentration of 1:100 and 1:25, respectively. The following day, samples were rinsed with five 30 min washes in BT and incubated overnight with their respective secondary antibody (goat anti rabbit IgG Alexa Fluor 488, Invitrogen, or goat anti mouse Cy™5-linked, GE HealthCare Uk Ltd.), diluted at 1:1000 in PBT with 1% GS. Alternatively, samples were labeled with either Rhodamine phalloidin or Alexa Fluor 488 phalloidin (1:40, Invitrogen) diluted in PBST for 1 h at room temperature, followed by three 15 min washes in PBST and stored in 50% glycerol/50% PBS. Embryos were mounted to view in a lateral position on glass slides in 100% glycerol or Dako (Dako Sweden AB, Stockholm Sweden). Images from whole mount embryos were acquired using a Zeiss META 510 confocal microscope (excitation 543 nm, emission 560–615 band pass filter, using 20 or 40x lenses) and z-stack images were acquired using a Leica (Leica SP5). Alexa 488 labeled specimens were excited with 488 nm laser line and emitted light was collected at 500–550 nm band pass filter using 60x lens. For a comparative approach images were acquired from somites that were positioned in a region aligning with the yolk extension, immediately below the point of attachment with the yolk ball.

### Muscle fiber and myofilament structure

The length of myofibrils was obtained by tracing lines along them using the “open free shape curve drawing mode” (Imaris software, Bitplane, Oxford Instrumentals). In addition end–end length measurements (i.e., between myosepta in the trunk muscles) were taken from a straight line that connected the starting and end points of the curved line. The myofibril length was normalized to the end-end length as an index for straightness, a measurement used as an indicator of alignment. The width of the myofibrils was taken perpendicularly to the median plan of the somite which was first established by measuring the width of the somite at several levels and joining the center of these measurements. The length of the actin myofilaments was estimated by measuring between the boundaries of adjacent H zones (H-H measurement, **Figures 5A,B**), five measurements were taken per fish.

### Larval motility assay

Swimming basal behavior was assessed by recording the velocity of both control and Tricaine treated embryos over 2 min trials at 3, 5, 7, and 30 dpf using EthovisionTM. Larvae were filmed from above for 2 min, allocated into 1-s bins. Distance traveled per unit time (mm/s) and rotations (n/s) were assessed as a function of age (4-stages: 3, 5, 7, and 30 dpf) and Tricaine treatment (2-levels: Tricaine treated vs. control). Distance traveled data were entered into a random intercept linear mixed effects model, with individual ID nested in batch as a random effect. Denominator degrees of freedom were estimated using the Satterthwaite approximation. Initial null model fits indicated that the errors were non-normal, but this was corrected by a log (base-10) transformation. Rotations data were overdispersed (zero-inflated) and fitted to a mixed effects model with a negative-binomial distribution specified (generalized linear mixed effects model). As before, individual ID nested in batch was added as a random effect. The contribution of “time” to the variance was assessed by likelihood ratio test from the null models, but this was omitted from final models. This analysis was performed using the R “lme4” package.

### Statistical analysis

Statistical analysis performed with the Graphpad Prism, SPSS and R software. All analyses were performed with respect to a null-hypothesis rejection level of alpha = 0.05.

## Results

### Effects of pharmacological and genetic paralysis on myofibril organization

The appearance of the first locomotor behavior in the zebrafish embryo at 17 hpf, an alternating side-side contraction of the body, occurs as functional neuromuscular connections start to form (Eisen, 1991; Devoto et al., 1996; Saint-Amant and Drapeau, 1998; Brennan et al., 2005). The present study applied pharmacological paralysis and examined an immotile mutant line (*cacnb1*^*ts25*^*, relaxed*) to explore whether early active contractions have a role in the organization of myofibrils. The *cacnb1*^*ts25*^*, relaxed* mutant line carries a G-A non-sense point mutation in exon 13 of the gene CACNB1 which encodes the DHPR β1a subunit, leading to premature termination at the position Trp454. As the defect lies within the excitation-contraction coupling pathway, the embryos are immotile and initially alive but die at around 7–10 dpf.

**Figure 1:**
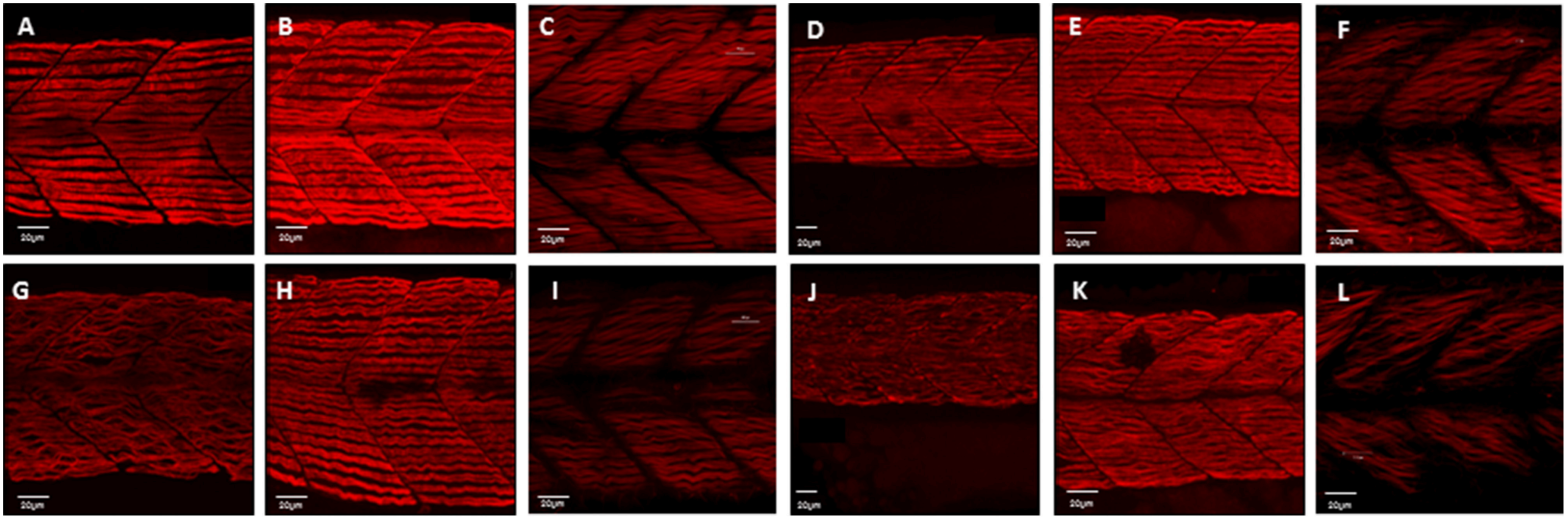
**Disruption of myofibril organization during paralysis is reversed by restoring movement in developing skeletal muscle**. Control embryos (Embryo Medium alone) and treated embryos (Embryo Medium with Tricaine) were incubated for 7 h starting at 17 hpf. At 24 hpf embryos were removed and control **(A)** and treated embryos **(G)** were fixed and stained immediately or put into recovery (Embryo Medium without Tricaine) and **(B)** control and **(H)** recovered embryos fixed and stained at 42 hpf. Some embryos were kept in recovery from 24 hpf up to 5 dpf before fixation and staining of both control **(C)** and recovered larvae **(I)**. *Relaxed* immotile mutants were fixed and stained at 24 hpf **(J)**, at 42 hpf **(K)**, and 5 dpf **(L)** as well as motile control siblings at 24 hpf **(D)**, 42 hpf **(E)** and 5 dpf **(F)**. Anti-myosin antibody (F59) in **(A,B,D,E,G,H,J,K)**, and Rhodamine phalloidin actin labeling in **(C,I,F,L)** reveals slow muscle fibers. For consistency, the somites imaged were taken at the level where the yolk sac and the yolk sac extension join. Scale bars corresponds to 20 μm.

In zebrafish there are separate populations of slow and fast muscle types which develop separately. By 24 hpf, slow muscle fibers have formed as superficial monolayer in the surface of the somite and are easy to visualize using immunohistochemical staining (Devoto et al., 1996). Early paralysis (between 17 and 24 hpf) led to myofibril disruption with the appearance of the “wavy” myofibril phenotype in immotile embryos, both the chemically paralyzed (Tricaine treated) and the immotile mutant larvae (*cacnb1*^*ts25*^*, relaxed*) (Figures 1G,J), in comparison to the control embryos (Figures 1A,D). A partial structural recovery of myofibril organization was observed in embryos that had been allowed to recover movement in Embryo Medium up until 42 hpf, after an initial chemical paralysis of 7 h administered between 17 and 24 hpf (Figures 1B,H). The structural recovery was maintained at 5 dpf (Figures 1C,I) whereas the myofibrils in immotile *relaxed* mutant remained wavy (Figures 1E,F,K,L).

**Figure 2:**
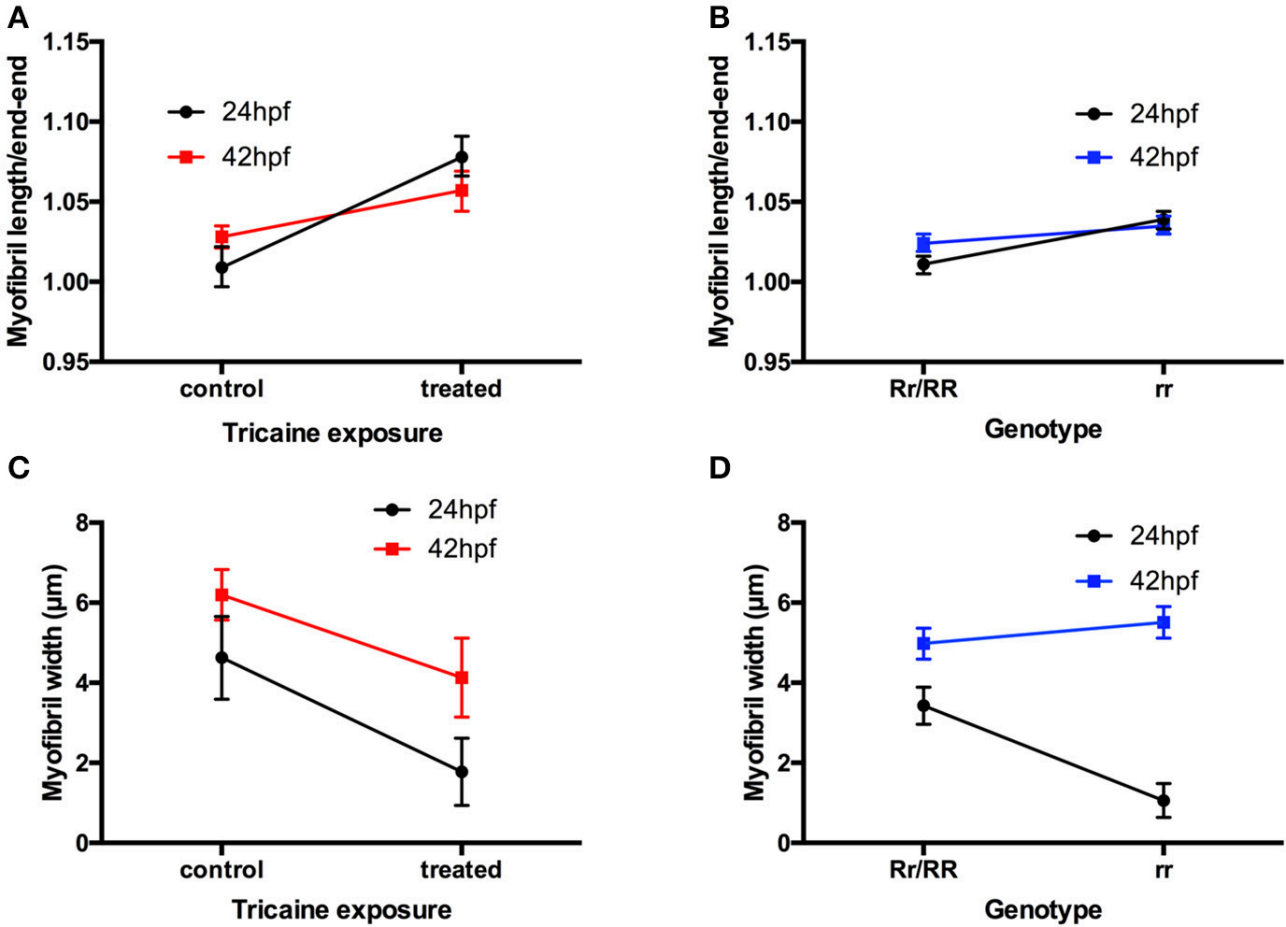
**Disruption of myofibril organization during paralysis is reversed by restoring movement in developing skeletal muscle. (A)** A factorial ANOVA with age (2-levels: 24 and 42 hpf) and treatment (2-levels: control and treated) was carried out on the ratios of myofibril length to end-to-end measurement. This revealed significant main effect of treatment, *F*_(1, 32)_ = 77.45, *p* < 0.001, but no main effect of age, *F*_(1, 32)_ < 1. There was, however, an age × treatment interaction, *F*_(1, 32)_ = 13.27, *p* < 0.01. As is clear from **(A)**, this interaction is characterized by a steeper increase in the ratio for treated individuals at 24 hpf than at 42 hpf. (length: controls *n* = 6, treated *n* = 6 and width: controls *n* = 17, treated *n* = 36). **(B)** A factorial ANOVA with age (2-levels: 24 hpf and 42 hpf) and genotype (2-levels: Rr/RR and rr) was carried out on the ratios of myofibril length to end-to-end measurement. This revealed significant main effect of genotype, *F*_(1, 116)_ = 49.81, *p* < 0.001, but no main effect of age, *F*_(1, 116)_ = 3.46, *p* = 0.07. There was, however, an age × genotype interaction, *F*_(1, 116)_ = 9.42, *p* < 0.01. As is clear from **(B)**, this interaction is characterized by a steeper increase in the ratio for homozygous individuals at 24 hpf than at 42 hpf. (length: controls *n* = 18, treated *n* = 6, width: controls *n* = 57, treated *n* = 23). **(C)** A factorial ANOVA with age (2-levels: 24 and 42 hpf) and treatment (2-levels: control and treated) was carried out on the width of myofibrils. This revealed significant main effect of treatment, *F*_(1, 129)_ = 30.06, *p* < 0.001, and of age, *F*_(1, 129)_ = 19.08, *p* < 0.001. There was no age × treatment interaction, *F*_(1, 129)_ < 1. As is clear from **(C)**, Tricaine-treated embryos showed robust reductions in myofibril width. In addition, older embryos (42 hpf vs. 24 hpf) had wider myofibrils, regardless of Tricaine treatment. (length: Rr/RR *n* = 5, rr *n* = 5 and width: Rr/RR *n* = 10, rr *n* = 10). **(D)** A factorial ANOVA with age (2-levels: 24 and 42 hpf) and genotype (2-levels: Rr/RR and rr) was carried out on the width of myofibrils. This revealed significant main effects of genotype, *F*_(1, 478)_ = 200.06, *p* < 0.001, and of age, *F*_(1, 116)_ = 18.76, *p* < 0.001. There was also an age × genotype interaction, *F*_(1, 478)_ = 46.79, *p* < 0.001. As is clear from **(D)**, this interaction is characterized by a steeper increase in the width of myofibrils in homozygous individuals at 24 hpf than at 42 hpf. (length: Rr/RR *n* = 5, rr *n* = 5 and width: Rr/RR *n* = 13, rr *n* = 13).

The effect of paralysis on myofibril length and end-end length (as shown in Table 1, data used to calculate index for straightness) and width was assessed in Tricaine treated (17–24 hpf) embryos at 24 hpf and after recovery of movement at 42 hpf. Results showed that early paralysis (between 17 and 24 hpf) does affect both the myofibril length/end-end ratio and the width, whereas these measurements showed a recovery close to control values by 42 hpf [Figures 2A,C, factorial ANOVA with age (2-levels: 24 and 42 hpf) and treatment (2-levels: control and treated)]. Similar results were observed in *relaxed* immotile mutant [Figures 2B,D, factorial ANOVA with age (2-levels: 24 and 42 hpf) and treatment (2-levels: control siblings (RR/Rr) and *relaxed* immotile mutants (rr)]. In conclusion, the initial contractile events prior to 24 hpf are important in driving the early structural organization of the myofibrils. However, as recovery can occur independently of contraction by 42 hpf movement does not seem as critical for the organization of myofibril structure (myofibril length/end-end ratio and the width) during this later stage.

**Table 1:** **Myofibril length and end-end data in wildtype, Tricaine treated and mutant (*relaxed*) fish at 24 and 42 hpf**.

	**Myofibril length in wild-type fish (μm)**				**End-End length in wild-type fish (μm)**			
	**24 hpf**		**42 hpf**		**24 hpf**		**42 hpf**	
	**Control**	**Treated**	**Control**	**Recovered**	**Control**	**Treated**	**Control**	**Recovered**
Average	66.95	73.37	71.33	79.35	66.33	68.05	69.38	74.97
Number	6	6	18	6	6	6	18	6
SE	1.16	1.74	0.92	1.44	1.21	1.57	0.85	1.39
	**Myofibril length in** ***relaxed*** **fish (μm)**				**End-End length in** ***relaxed*** **fish (μm)**			
	**24 hpf**		**42 hpf**		**24 hpf**		**42 hpf**	
	**RR/Rr**	**rr**	**RR/Rr**	**rr**	**RR/Rr**	**rr**	**RR/Rr**	**rr**
Average	61.10	67.19	75.64	89.84	60.47	64.69	73.77	86.83
Number	30	30	30	30	30	30	30	30
SE	0.89	1.01	0.87	0.90	0.91	0.97	0.85	0.91

### Recovery of swimming behavior after pharmacological inhibition of embryonic movement

**Figure 3:**
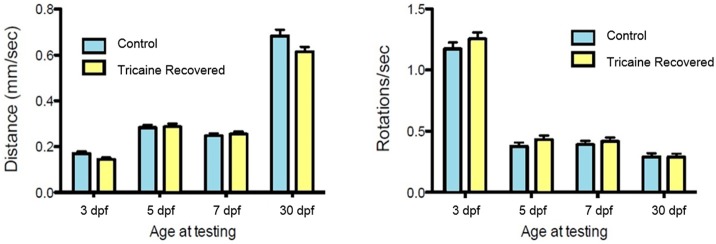
**Swimming behavior analysis**. Tricaine recovered fish swimming behavior, distance and rotations, was assessed at 3, 5, 7 days, and 30 dpf. No differences between treated and control fish were observed.

Recovery of movement after chemical immobilization was associated with a slow rescue of myofibril structure that occurred over several days. We examined whether this early paralysis and subsequent recovery affected swimming behavior at 3 dpf and beyond. Fish were allowed to recover after Tricaine immobilization as described above and their swimming behavior (i.e., distance covered and rotation frequency) was tested at 3, 5, 7 dpf and 30 dpf in comparison with non-immobilized controls. No difference in swimming behavior was observed between controls and the group treated with Tricaine at 3 dpf and thereafter (Figure 3) showing that the larvae can recover motile function after an initial period of immobilization and myofibril disorganization.

### Effects of paralysis and recovery on the length force relationship

**Figure 4:**
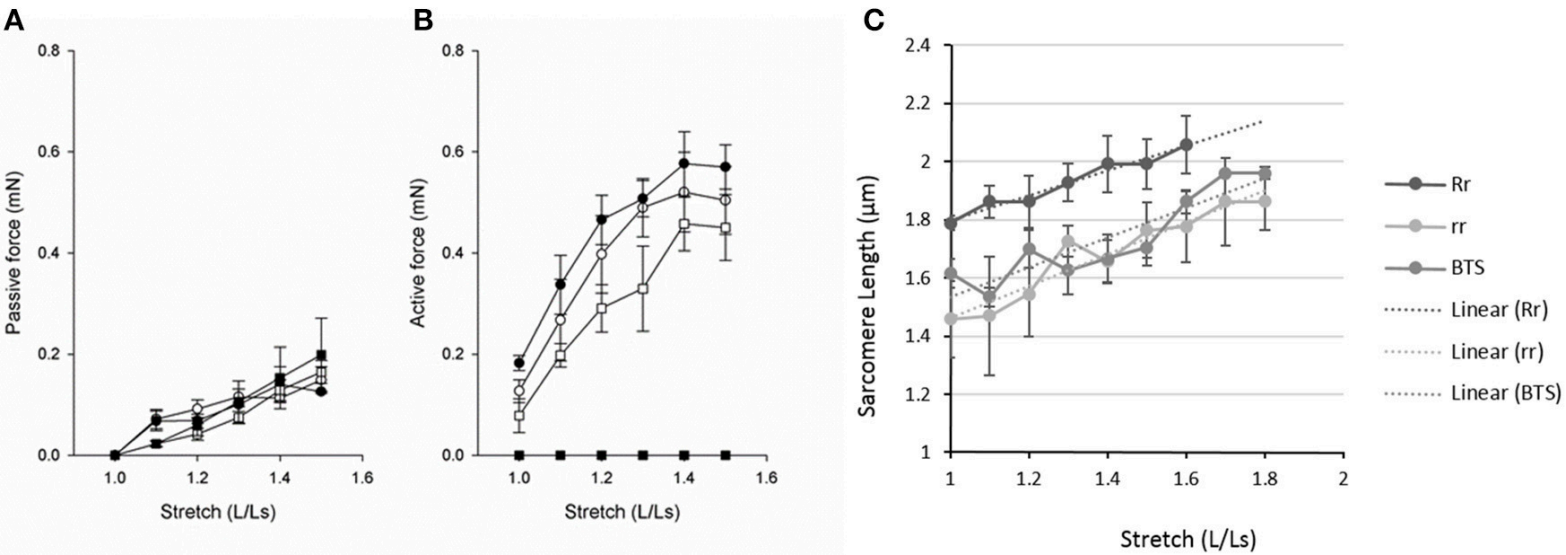
**Passive force (A), active force (B) and sarcomere length (C) at different lengths (L) relative to slack length (Ls)**. **(A,B)** Open circles, 7 dpf larvae held in Embryo Medium; filled circles, 7 dpf larvae held in Embryo Medium after Tricaine immobilization between 17 and 24 hpf; open squares, control siblings of the *relaxed* strain and filled squares, *relaxed* immotile mutants (*n* = 3–8). **(C)** Control siblings (Rr, dark gray), mutants (rr, light gray), and Tricaine-BTS treated larvae (BTS, mid gray). We observed that at stretches above 60% the controls siblings tended to break. The Tricaine-BTS and *relaxed* mutant larvae were possible to stretch significantly further without breaking, up to 80%. However, the sarcomere length did not increase further above 70%.

The mechanical function of Tricaine immobilized and recovered larvae and immotile *relaxed* mutants were examined by mounting larval muscle preparations for force recordings. The length of the samples (L) was increased in steps from slack length (Ls) and at each relative stretch (L/Ls) passive and active force were recorded. As seen in Figure 4A passive force increased as the muscles were stretched. The preparations were stretched to a length above the optimal length (Lopt), i.e., where active force was maximal. The passive force-stretch relationships of the larvae that had been immobilized by Tricaine between 17 and 24 hpf and allowed to recover until 7 dpf were similar to that of the controls. Also, the length-passive force relationship of the mutant *relaxed* larvae did not differ from their control siblings at 5 dpf. Figure 4B shows the active force in the different groups. The Tricaine treated larvae were not different from their controls at 7 dpf and had similar length active force relationships with an optimal length at about 1.4 L/Ls, showing that the contractile function is fully established after the initial period of immobilization. The *relaxed* mutant larvae did not react to electrical stimulus and did not develop any active tension. We also attempted stimulation using high K^+^ concentration (80 mM) or Caffeine (20 mM) in the intact *relaxed* mutant larvae but this did not result in any active tension.

In a separate set of experiments, we measured sarcomere length (SL) at different degrees of relative stretch (L/Ls) in immotile *relaxed* mutants (rr) compared to control siblings (Rr/rr) at 5 dpf (Figure 4C). The results show that the sarcomere length increased linearly against stretch in each groups (as shown in Figure 4C). Furthermore, individual sarcomere stretch is proportional to that of whole muscle in all groups (with a correlation coefficient for control siblings of *r* = 0.9808, for *relaxed* mutants of *r* = 0.9631 and for Tricaine-BTS treated larvae of *r* = 0.9024). However, sarcomere length was significantly shorter at all degrees of stretch in both the immotile *relaxed* mutants and Tricaine-BTS treated larvae at 5 dpf compared to the motile control siblings (Figure 4C). Furthermore, the sarcomere elasticity of both immotile *relaxed* mutant and Tricaine-BTS treated larvae, allowed an additional 20% stretch compared to the motile control siblings. Using the sarcomere length data (Figure 4C) and the stretch that produces the maximum force (Lopt, as measured from the active force experiments, Figure 4B), optimal sarcomere length was determined. Optimal sarcomere length was estimated to be 1.99 ± 0.10 μm in control siblings which is similar to that obtained for control wildtype (2.1 μm, (Li et al., 2013); whereas sarcomere length in the *relaxed* mutant was found to be significantly shorter (1.66 ± 0.08 μm). The relationship between sarcomere length and active force is influenced by the interaction between thick and thin filaments, and a difference in thick filament structure will influence the shape of the relationship. Although data on sarcomere structure in zebrafish is available (Squire et al., 2008; Ha et al., 2013), the detailed structure of the larval thick filaments is not known. The larval muscles are multicellular and tend to break at lengths above optimal, making demonstration of the plateau and the descending limb of the length-force relationship difficult (Dou et al., 2008). Thus, the interpretation of filament interaction based on mechanical data is complex. It is also noted that the ascending limb of the length-active force relationship is comparatively steep, which might reflect that activation mechanisms, in addition to filament overlap, determine active force at this length range.

### Effects of paralysis and recovery on thin filament length and interfilament spacing

**Figure 5:**
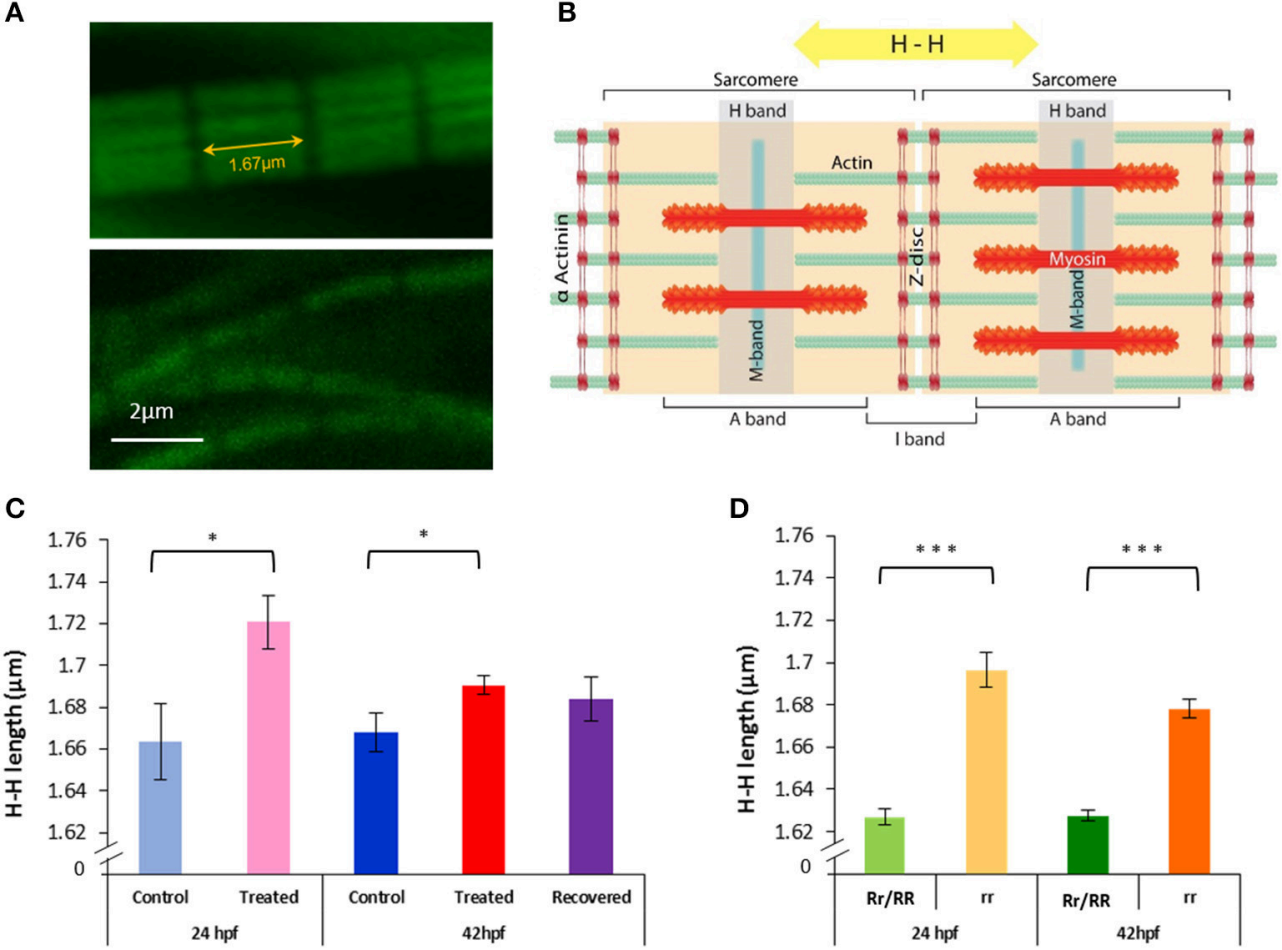
**Paralysis leads to actin lengthening in both *relaxed* mutant and Tricaine treated embryos, subsequent movement restoration in recovered embryos leads to actin length complete rescue**. **(A,B)** H-H measurement. Confocal images of actin filaments stained with phalloidin (1/40) were taken (upper panel 24 hpf control embryo and lower panel 24 hpf Tricaine treated embryo) and measurements between H bands, across Z discs and sarcomeres, were made. **(C)** 17–24 hpf paralysis caused a significant lengthening of actin filaments H-H at 24 hpf (^*^p < 0.05) (*n* = 20 in 5 controls, *n* = 15 in 3 treated embryos, *t*-test). At 42 hpf, recovered embryos (17–24 hpf paralysis) showed a full recovery (*n* = 50 in 10 control embryos, *n* = 40 in 8 recovered embryos, unpaired *t*-test), in contrast with treated embryos (17–42 hpf paralysis) which did not recover (*n* = 50 in 10 control embryos, *n* = 135 in 27 treated,^*^p < 0.05). **(D)** Immotile *relaxed* mutants, rr, showed a very significant lengthening of actin filaments H-H at 24 hpf (^***^p < 0.001) (*n* = 115 in 23 immotile *relaxed* mutants rr, *n* = 120 in 24 motile control siblings Rr/RR and at 42hpf (^***^p < 0.001) (*n* = 105 in 21 immotile *relaxed* mutants rr, *n* = 135 in 27 motile control siblings Rr/RR). Interestingly, in contrast with the Tricaine treated immotile embryos, the immotile *relaxed* mutant (rr) actin is still very significantly longer than the motile control siblings (Rr/RR) actin at 42 hpf. It is noticeable that in the complete absence of movement up to 42 hpf actin filaments showed a significant shortening between 24 and 42 hpf in between both treated and control embryos and immotile *relaxed* mutants and motile control siblings, respectively (*n* = 15 in 3 24 hpf treated, *n* = 135 in 27 42 hpf treated embryos, ^*^p < 0.05 unpaired *t*-test; and *n* = 115 in 23 24 hpf immotile *relaxed* mutants rr, *n* = 105 in 21 42 hpf immotile *relaxed* mutants rr, ^*^p < 0.05 unpaired *t*-test).

Given the observations that paralysis alters myofibril arrangement and shortens sarcomere length in developing zebrafish embryos, actin remodeling was assessed by measuring actin myofilament length during paralysis at 24 and 42 hpf and after movement recovery at 42 hpf, in both Tricaine treated and immotile *relaxed* mutant embryos. For this purpose, we estimated the thin filament length by determining the distance between the H zones in the sarcomeres in Alexa488 phalloidin stained larvae (Figures 5A,B).

Immotile *relaxed* mutants showed a very significant lengthening of actin filaments at 24 and 42 hpf in comparison to motile control siblings (Figure 5D). Tricaine paralysis also caused a significant lengthening of actin filaments at 24 and 42 hpf (17–42 hpf paralysis), in comparison to motile controls (wildtype) (Figure 5C). This implies that inhibition of movement causes significant lengthening of actin filaments in the developing skeletal muscle. It should also be noted that there was a significant shortening of actin filaments in heterozygote mutant control siblings compared to control (wildtype) fish at 24 hpf (1.627 ± 0.004 in control siblings and 1.664 ± 0.018 in control fish, ^**^p < 0.01) and at 42 hpf (1.628 ± 0.003 in control siblings and 1.668 ± 0.009 in control fish ^***^p < 0.001, unpaired *t*-test). Furthermore, 42 hpf recovered embryos (17–24 hpf paralysis followed by movement recovery) showed a rescue of actin length when compared to controls (Figure 5C). This suggests that movement contributes to the regulation of actin filament length. However, it is also interesting to note that in embryos that remained paralyzed throughout the experiment (17–42 hpf), both *relaxed* mutant and paralyzed embryos, showed a small but significant shortening in actin filament length between 24 and 42 hpf (rr: 1.697 ± 0.008 at 24 hpf and 1.678 ± 0.004 at 42 hpf, ^*^p < 0.05, treated: 1.721 ± 0.013 at 24 hpf and 1.691 ± 0.005 at 42 hpf, ^*^p < 0.05, unpaired *t*-test). This indicates that an additional component of the mechanism regulating actin length is independent of movement.

**Figure 6:**
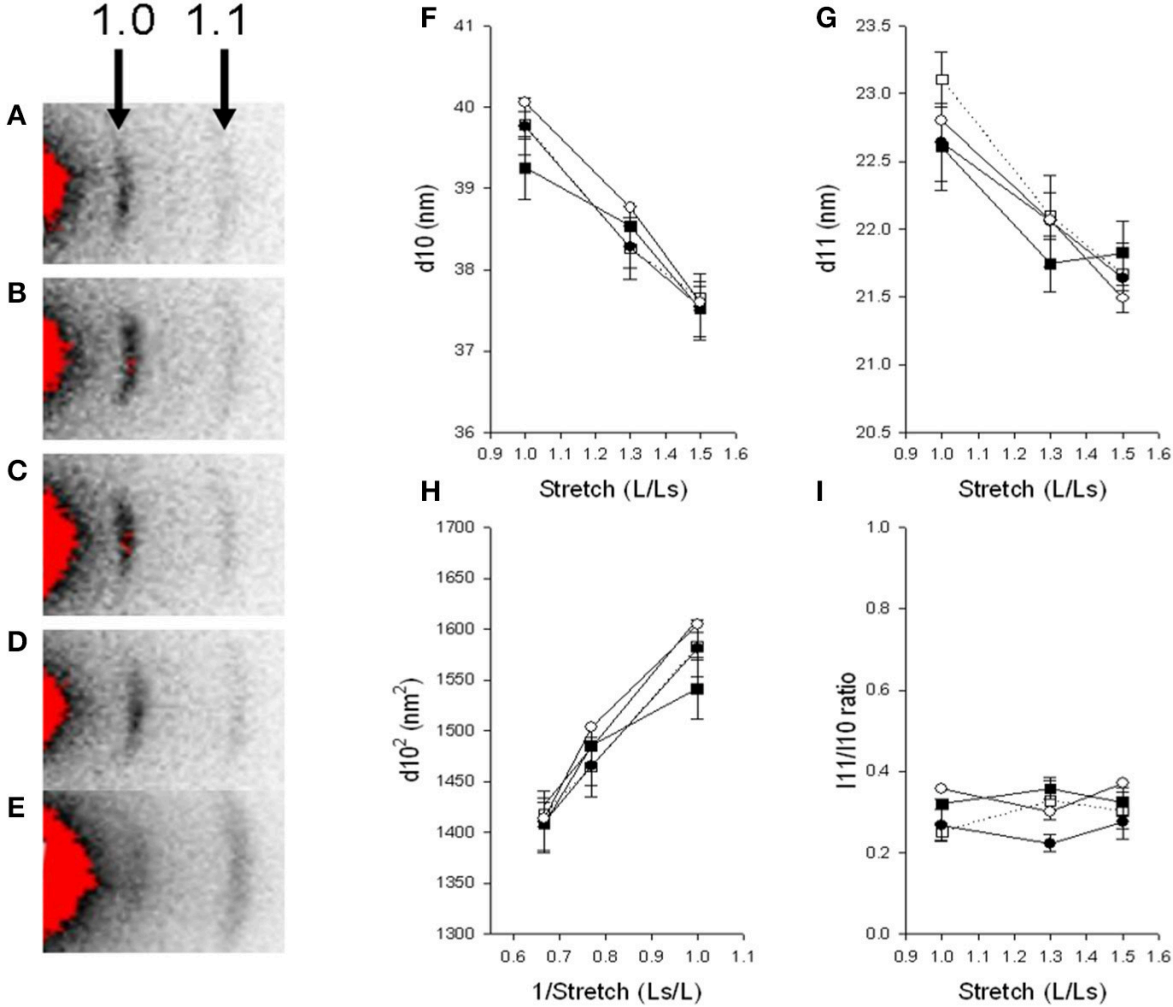
**Small angle X-ray diffraction**. *Relaxed* immotile mutants (**D**, Filled circles), their control siblings (**C**, open circles) at 6 dpf, larvae treated with Tricaine between 17 and 24 hpf and recovered until 6 dpf (**B**, filled squares) and their controls kept in Embryo Medium until 6 dpf (**A**, open squares). Panels **(F,G)** shows the spacing (d10, d11) of the 1.0 and 1.1 reflections at different degrees of stretch, **(H)** shows the relationship between d1.0^2^ and inverted value of stretch. Panel **(I)** shows the intensity ratio of the 1.1 and 1.0 reflection at different degrees of stretch. *n* = 4–6 except in the controls kept in Embryo Medium until 6 dpf, where *n* = 1. Panel **(E)** shows a *relaxed* immotile mutant sample in rigor, displaying an increase in the outer 1.1 reflection, the 1.1/1.0 intensity ratio was 3.67 compared to about 0.4 in samples under normal conditions **(D,I)**.

Interfilament spacing was measured in 5 dpf muscle preparations of the immotile *relaxed* mutants and their control siblings, to explore if the complete lack of contractile activity during development, and the longer thin filament lengths (as determined at 2 dpf) affect the lateral arrangement of myofilaments in the sarcomere. We also examined the filament structure in larvae recovered after Tricaine immobilization 17–24 hpf in comparison with their controls at 5 dpf. For these experiments, we used small-angle X-ray diffraction and determined the lattice spacing from the 1.0 and 1.1 equatorial reflections (Figure 6). The equatorial 1.0 and 1.1 reflections were clearly visible in all groups (Figures 6A–E), showing that even in the completely immotile *relaxed* larvae a regular filament lattice is assembled (Figures 6D,E). X-ray patterns were recorded at different degrees of stretch and the spacing of the equatorial reflections evaluated (Figures 6F,G). Stretch of the muscles resulted in an outward movement of the reflections reflecting shrinkage of the filament lattice due to constant volume behavior in all groups. The spacing of the 1.0 and 1.1 reflections (d10 and d11) were similar in the four groups at all degrees of stretch. In Figure 6H, d10^2^ is plotted against the inverted stretch which resulted in a linear relationship. Since the degree of stretch was linearly related to sarcomere length we could then estimate sarcomere volume from the slope of the relationship between d10^2^ and 1/sarcomere length (Millman, 1998). In control siblings sarcomere volume was measured at 3.36 × 10^−3^ μm^3^, which is similar to the values found in other muscle studies; whereas *relaxed* mutants showed a 15% lower volume of approximately 2.89 × 10^−3^ μm^3^. We examined the intensity ratio of 1.0 and 1.1 reflections. The inner 1.0 reflection was strong with a low 11/10 intensity ratio of about 0.2–0.4 in all groups, reflecting that cross bridges are positioned close to the thick filament backbone with no major differences between the examined groups.

Since the mechanical experiments showed that the immotile *relaxed* larvae were not able to develop active force, due to an activation failure, we examined if myosin cross bridges can attach to actin by introducing a rigor state in the *relaxed* group. Larvae were anesthetized and examined after 2.5 h in Embryo Medium in the presence of 5 mM NaCN. As seen in Figure 6E, this resulted in a marked change in the intensity ratio with an increase in the 1.1 intensity and a relative weakening of the 1.0, demonstrating that a state of rigor could be achieved in the *relaxed* mutant larvae. On average the intensity ratio in rigor was 2.4 ± 0.3 (*n* = 4) in control siblings and 2.7 ± 0.1 (*n* = 2) in the *relaxed* immotile mutants.

### Effects of paralysis on the localization of tropomodulins

Given that actin filament elongation was observed in immotile embryos, we examined the expression of Tropomodulin 1 (Tmod1) and Tropomodulin 4 (Tmod4) in both *relaxed* mutant and chemically treated embryos to determine whether their localization was disrupted, compared to control siblings and wild-type, respectively.

**Figure 7:**
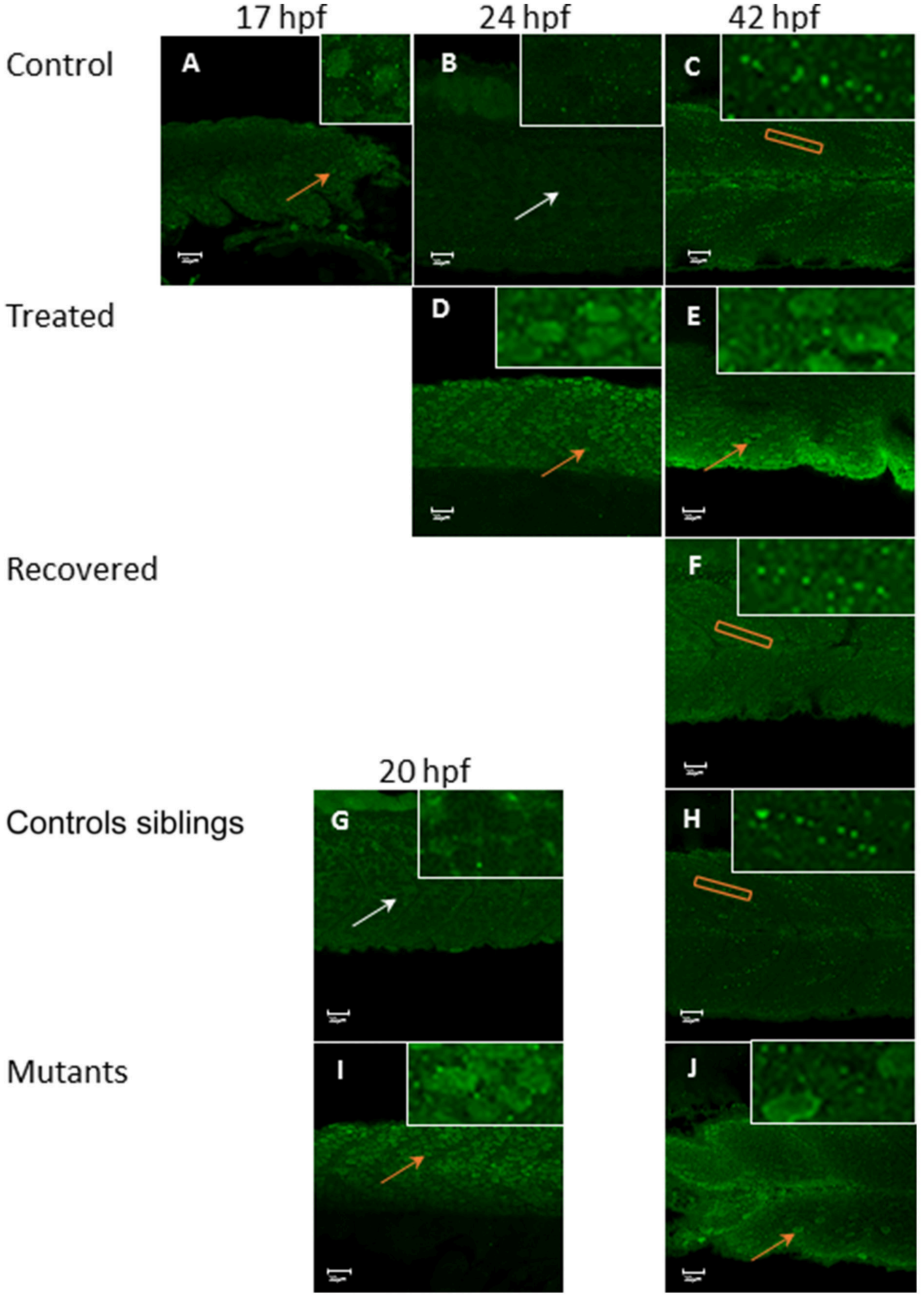
**Tropomodulin 1 localization within the fast skeletal muscle from 17 hpf up to 42 hpf in immotile and control embryos**. In control embryos **(A–C)** Tmod1 is initially located in the nucleus of the fast muscle cell at 17 hpf (orange arrow, inserts) **(A)** and then migrate into the skeletal muscle cell cytosol at 24 hpf (empty nuclei, white arrow, insert) **(B)** where it stays and get organized linearly by 42 hpf (orange rectangle, insert) **(C)**. Tricaine treated immotile embryos **(D,E)** display a nuclear localization of Tmod1 at both 24 hpf **(D)** and 42 hpf **(E)** (orange arrows, inserts). Recovered motile embryos **(F)** display at 42 hpf a similar cytosolic alignment of Tmod1 as control embryos (orange rectangle, insert). Controls siblings motile embryos **(G,H)** display a cytosolic pattern of Tmod1 from 20 hpf **(G)** (empty nuclei, white arrow, inserts) which is linearized by 42 hpf **(H)** (orange box, insert) as control and recovered embryos. Immotile *relaxed* mutants embryos **(I,J)** display a similar nuclear localization of Tmod1 at both 20 hpf **(I)** and 42 hpf **(J)** as Tricaine treated embryos (orange arrows, inserts). Right hand corner inserts shown at a magnification of X5 compared to main image.

In control embryos, Tmod1 is located in the nucleus of skeletal muscle fibers prior to movement at 17 hpf (Figure 7A) but by 24 hpf the distribution of the protein has changed, it was less prominent in the nucleus and was mainly found in the cytosol (Figure 7B). By 42 hpf the staining appears punctate and arranged in lines within the cytoplasm of the fibers themselves (Figure 7C). In contrast to control embryos, Tricaine treated immotile embryos display nuclear localization of Tmod1 at both 24 hpf (Figure 7D) and 42 hpf (Figure 7E). Whereas, in recovered motile embryos (Figure 7F) at 42 hpf, Tmod1 displays a similar cytosolic staining pattern to control embryos. In summary, prior to movement (at 17 hpf) and in immotile embryos (at 24 and 42 hpf) Tmod1 staining is observed at the nucleus, whilst in motile embryos it is located in the cytoplasm. These observations were investigated further using the immotile *relaxed* mutant line. Control siblings motile embryos display cytosolic Tmod1 staining at 20 hpf (Figure 7G) and had a similar patterns to control and recovered embryos by 42 hpf (Figure 7H). Mutant immotile *relaxed* embryos display a nuclear localization of Tmod1 at both 20 hpf (Figure 7I) and 42 hpf (Figure 7J), similar to that observed in Tricaine treated immotile embryos. In summary these results suggest that the relocation of Tmod1 from the nucleus to the cytoplasm is driven by embryonic active movement.

**Figure 8:**
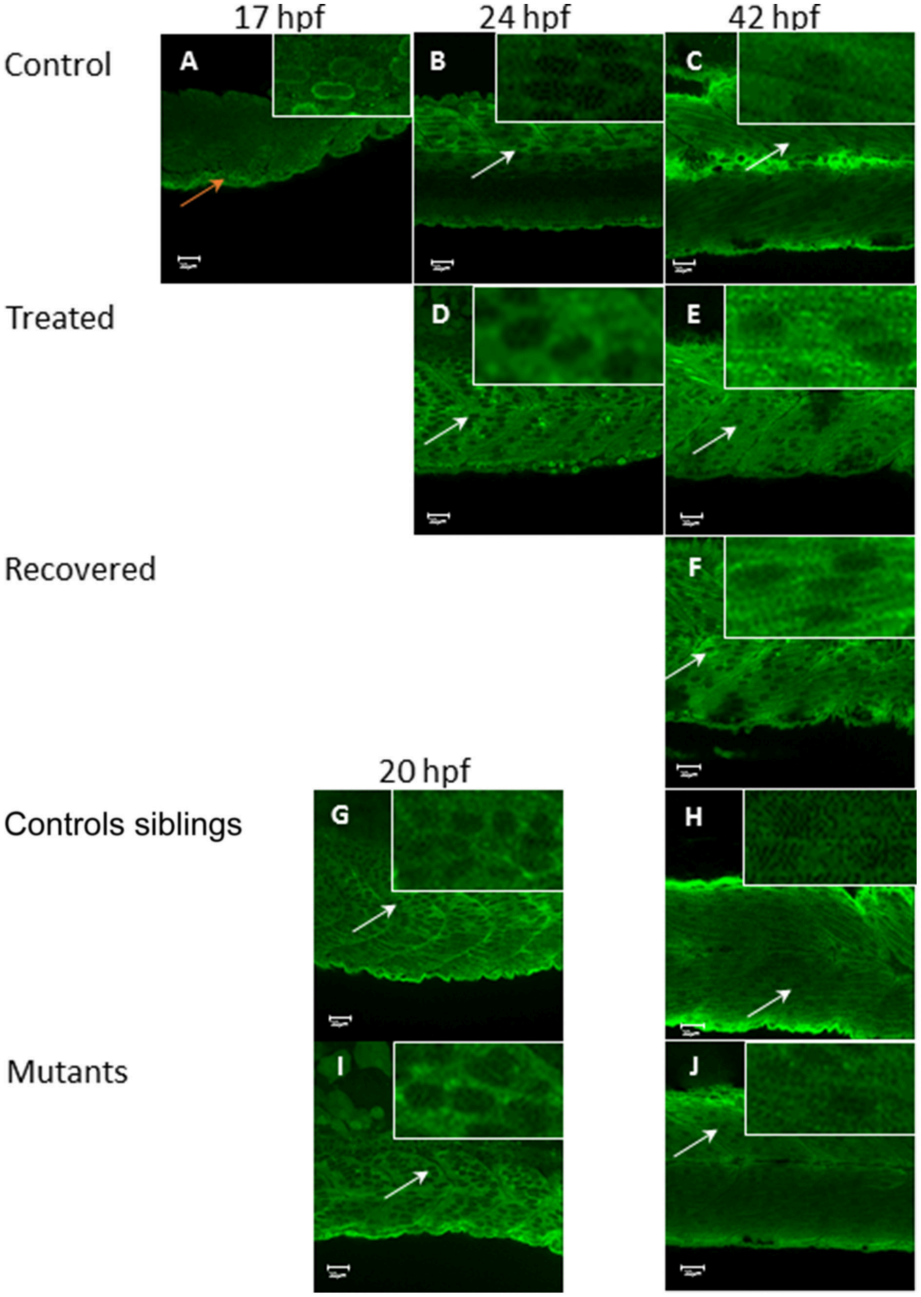
**Tropomodulin 4 localization within the fast muscle cell from 17 hpf up to 42 hpf in immotile and control embryos**. In control embryos **(A–C)** Tmod4 is initially located in the nucleus of the fast muscle cell at 17 hpf (orange arrow, insert) **(A)** and then migrate into the fast muscle cell cytosol at 24 hpf **(B)** where it stays up to 42 hpf (empty nuclei white arrows, inserts) **(C)**. Tricaine treated immotile embryos **(D,E)** display a cytosolic localization of Tmod4 at both 24 hpf **(D)** and 42 hpf **(E)** (empty nuclei, white arrows, inserts). Recovered motile embryos at 42 hpf **(F)** display a similar cytosolic localization of Tmod4 as control and treated embryos (empty nuclei, white arrows, inserts). Controls siblings motile embryos **(G,H)** display a cytosolic localization of Tmod4 from 20 hpf **(G)** which is maintained up to 42 hpf **(H)** (empty nuclei, white arrows, inserts). Immotile *relaxed* mutants embryos **(I,J)** display a similar cytosolic localization of Tmod4 at both 20 hpf **(I)** and 42 hpf **(J)** as control siblings (empty nuclei, white arrows, inserts). Right hand corner inserts shown at a magnification of X5 compared to main image.

In control embryos, Tmod4 is located initially in the nucleus of the muscle cell at 17 hpf (Figure 8A), but by 24 hpf (Figure 8B) the distribution of the protein has changed with a stronger signal in the cytosol, where it remains at 42 hpf (Figure 8C). In Tricaine treated immotile embryos, Tmod4 displays a cytosolic localization from 24 hpf onwards (Figures 8D,E) and recovered motile embryos (Figure 8F) also display cytosolic staining of Tmod4 at 42 hpf. Both the motile control siblings and the immotile *relaxed* mutant embryos display Tmod4 within the cytosol from 20 hpf onwards (Figures 8G–J). In summary, these results suggests that the relocation of Tmod4 from the nucleus to the cytoplasm is unaffected by embryonic movement.

## Discussion

Inhibition of contraction between 17 and 24 hpf caused a significant increase in length and decrease in width of myofibrils. Furthermore, sarcomere shortening was also observed as a result of paralysis. Initial pharmacological paralysis did not affect the passive and active force of the muscle after recovery; however, active force could not be generated upon stimulation in *relaxed* embryos. Paralysis inhibits active tension, which recovers progressively once motility is restored, ultimately resulting in normal swimming behavior. Lattice spacing at the myofilament level was unaffected by both paralysis (*relaxed* mutants) and paralysis followed by recovery (Tricaine treatment). In the absence of contraction, a lengthening of the actin filaments was observed. However, the paralysis-induced elongation of actin at 24 hpf was shown to recover by 42 hpf. Examination of the capping proteins Tmod1 and 4 revealed that paralysis selectively affects the localization of Tmod1, which is retained in the myocyte nuclei, rather than in the cytosol. In contrast, the expression of Tmod4 was unaffected by paralysis and it underwent relocation from the nucleus to the cytosol. Overall, the results show that during zebrafish skeletal muscle development, the later steps of myofibrillogenesis are regulated by a combination of movement-driven and movement-independent events.

### Movement-dependent regulation of myofibril assembly

Actin is a key protein of the sarcomere thin filaments, which allow force transmission along the myofibrils. Results revealed that there is a lengthening of the actin filaments in the absence of contraction suggesting that actin filament length is modulated via contractile activity. This data fits with our observations at the myofibril level and suggests that movement-induced regulation of actin polymerization and thin filament length may be an underlying factor of myofibril organization, namely elongation during development. Evidence from other studies suggests that actin filament remodeling, specifically lengthening, may have a direct impact on myofibril organization (Gokhin and Fowler, 2013). Previous work has shown that myofilaments and sarcomere length are disrupted by paralysis (Brennan et al., 2005; Etard et al., 2005) and our observations of actin lengthening during paralysis are consistent with this idea.

The regulation of actin length was explored further by assessing the effect of paralysis on the localization of the actin-capping proteins Tmod1 and 4. Tmod1 and 4 function to promote polymerization and depolymerization of actin filaments within skeletal muscle (Littlefield and Fowler, 2008). Over expression of Tmod1 leads to a decrease in actin incorporation and shortening, whilst inhibition of Tmod1's actin pointed-end capping activity has the reverse effect (Gokhin and Fowler, 2011). It is argued that the normal role of Tmods in muscle fibers, alongside the antagonistic actions of the leiomodins, is to control length by inhibiting polymerization and lengthening of filaments (Nworu et al., 2015). Further, disruption of these Tmods has been linked to the pathogenesis of muscular dystrophy (Gokhin et al., 2014).

We observed an initial expression of Tmod1 in the nuclei of the muscle cells and subsequently a movement-dependent translocation to the cytosol. Our observations are supported by *in vitro* studies in mammalian skeletal muscle where Tmods were shown to be expressed both in the nucleus as well as the cytosol (Kong and Kedes, 2004). Furthermore, in immotile embryos Tmod1 is present in the nucleus but absent from the cytosol and this correlates with a significant lengthening of actin filaments. Taken together these observations suggest that in the absence of contractile activity, Tmod1 does not relocate to the cytosol and this results in the generation of longer actin filaments. In conclusion, the presence of Tmod1 in the cytosol may limit contraction-driven actin lengthening during sarcomere maturation in development. The movement-driven relocalization of Tmod1 observed in this study fits with the wider view of a mechanically-driven relocalization of sarcomeric proteins between different cellular compartments, a regulatory mechanism proposed to be important for controlling several aspects of cell function, including survival (Clark et al., 2002; Lange et al., 2006). Indeed there is evidence that nuclear Tmod plays a role in the proliferation and differentiation of cultured skeletal muscle cells (Kong and Kedes, 2004).

Tmod4, shows an early nuclei localization followed by translocation into the cytosol, which occurs simultaneously with Tmod1. The localization of Tmod4 was unaffected by paralysis and its cellular localization is not dependent on contraction. However, although Tmod4 is not regulated by contraction, its translocation still occurs concurrently with Tmod1 by 20 hpf, which suggests that another signal induces its transfer at this specific developmental stage. Therefore, the shortening of actin observed between 24 and 42 hpf in immotile embryos is very likely to be the result of the regulating action of Tmod4, as in these embryos Tmod1 is missing at the actin pointed ends. In contrast, actin shortening in the motile recovered embryos can be attributed to both Tmod1 and Tmod4, which are capping actin pointed ends. This could explain why the recovered embryos are the only one to restore actin length to its normal size, suggesting that both Tmod1 and 4 are required to complete this process. Recently Tmods have been shown to have further functional roles regulating actomyosin crossbridge formation and impacting on force production in skeletal muscle (Ochala et al., 2014). Therefore, Tmods relocalization from the nucleus to the cytosol may not only affect actin filament length but also influence muscle contractility directly.

This study showed that during normal skeletal muscle development, Tmod1 and 4 are located in the nuclei of skeletal muscle cells prior to the onset of embryo movement at 17 hpf. This initial nuclear localization could indicate additional roles for Tmods in the myocyte; for example, they might be interacting with actin filaments in this location. Similar interactions have been described in cardiac muscle where Tmods are associated at the plasma membrane before they locate to the sarcomeres (Chu et al., 2003; Ehler et al., 2004). Tmods, by controlling actin length, ultimately control the regular array of sarcomeres within the skeletal muscle myofibrils, leading to proper myofibril organization.

### Movement-independent regulation of myofibril assembly

The results showed that inhibition of contraction between 17 and 24 hpf causes a significant decrease in the width and a significant increase in the length of the myofibrils. Recovery of myofibril width and length occurred independently of movement by 42 hpf. This suggests that, from 24 hpf onwards, there is a movement-independent mechanism that regulates myofibril width and length during zebrafish embryonic skeletal muscle development. The proposal that there is a myofibril regulatory mechanism independent of movement is supported by a previous study in our laboratory that showed that the disruption of myofibril organization in chemically paralyzed embryos was milder when the drug was applied at later developmental stages, from 24 to 48 hpf (Lahne et al., 2009). At the myofilament level, the lattice spacing was found to be regulated independently of movement. Results also show that the paralysis-induced elongation of actin at 24 hpf appears to be partially recovered by 42 hpf, indicative of a regulatory component that occurs independently of contraction. Furthermore, X-ray data indicates that by 6 dpf, myofilament lattice spacing is not disrupted by paralysis. It is assembled and allows attaching of cross bridges even in immotile *relaxed* mutants which can reach a rigor state. This demonstrates that the actin and myosin myofilament lattice spacing is regulated independently of movement.

In this study, the active property of skeletal muscle was also examined in drug treated recovered larvae. Tricaine recovered larvae, paralyzed from 17 to 24 hpf, displayed an active force that was comparable to control values. Skeletal muscle active force hinges on the ability of myofilaments to form cross-bridges, and this in turn relies on the correct myofilament spacing. The organization of the myofilament lattice was shown to be a movement-independent process and thus, it could be argued that the potential of paralyzed muscle to generate active force is also independent of movement. This hypothesis is supported by the ability of immotile *relaxed* mutants to reach rigor when exposed to cyanide. Furthermore, in skinned *relaxed* mutant embryos, skeletal muscle contraction on one side of the truck can be elicited by application of Caffeine (Zhou et al., 2006). Thus, the ability of the skeletal muscle to generate active force is not determined by the initial spontaneous contractile event in zebrafish embryos. Our results show that the active properties of skeletal muscle is not disrupted by paralysis and correlates with the correct spacing of myofilaments.

In summary, myofibril organization in developing zebrafish skeletal muscle appears to be regulated by a combination of both contraction and contractile independent mechanisms. Thus, this study has demonstrated the existence of several pathways which regulate myofibril assembly and its subsequent maintenance during development *in vivo*. The challenge will be to determine the underlying mechanisms and the cellular signals that are involved in the different pathways. In addition, a deeper understanding of myofibril assembly pathways *in vivo* will ultimately help to inform study of human skeletal muscle disease and could potentially lead to the development of new therapies.

## Author contributions

RA, LM, MP, AA, and ML were all instrumental in designing at least some aspects of the work, interpretation of data, drafting and revising the manuscript. They have had final approval of the version to be published and agreement to be accountable for all aspects of the work in ensuring that questions related to the accuracy or integrity of any part of the work are appropriately investigated and resolved.

### Conflict of interest statement

The authors declare that the research was conducted in the absence of any commercial or financial relationships that could be construed as a potential conflict of interest.
